# Current Management of Neurological Wilson’s Disease

**DOI:** 10.5334/tohm.938

**Published:** 2025-05-05

**Authors:** V. H. Ganaraja, Vikram V. Holla, Pramod Kumar Pal

**Affiliations:** 1 Department of Neurology, National Institute of Mental Health and Neuro Sciences, Bengaluru, India-560029

**Keywords:** Wilson’s disease, Movement disorders, Treatment, Outcome, Neurological and/or hepatic WD

## Abstract

Wilson’s disease (WD) is a disorder of copper metabolism due to variants in the *ATP7B* gene. This autosomal recessively inherited disorder is characterized by the accumulation of copper in various body parts, mainly the liver, brain, and kidneys. Initially, WD was described to involve the hepatic and neurological systems. Subsequently, diverse presentations have been reported with skeletal and hematological manifestations and various constellations of symptoms. Neurological manifestations of WD are varied, ranging from asymptomatic neurological state to refractory dystonia. Earlier, the diagnosis was based only on measuring serum ceruloplasmin levels, urinary copper levels, and imaging. Advanced genetic testing has provided an additional mode of diagnosis in the patient, screening of the family members and, a way to better understand the genotype-phenotype associations of the disease if there are any. In the last few decades, the treatment of WD has evolved from symptomatic treatment and chelation therapy to many new advanced measures for both copper chelation and symptomatic relief. With a better understanding of the genetic aspects of WD in recent years, there has been more focus on gene therapy, novel therapies targeting ATP7B genes, and therapies targeting mutant proteins to prevent copper accumulation. This article highlights the advances in diagnostic methods and treatment modalities in WD.

## Introduction

Copper is an essential metal required as a cofactor for many metabolic pathways in the human body. The normal requirement of copper is about 1.5–2.5 mg/day. The copper in the diet is absorbed mainly from the duodenum and through the portal circulation and is stored in the liver. From the liver, it is utilized for metabolic needs and excess copper is excreted through bile. Impairment of this biliary excretion mechanism leads to the accumulation of excess copper in the liver with subsequent spillage into the systemic circulation. This excess copper gets deposited in various body parts, causing clinical manifestations. Wilson’s disease (WD) is a disorder of copper metabolism due to mutations in the *ATP7B* gene leading to abnormal copper accumulation [1]. Clinical manifestation of WD mainly depends on the site of copper deposition, leading to copper toxicity and organ dysfunction. The prevalence of this rare genetic disorder varies globally and is generally estimated to be between 1 in 30,000 to 1 in 40,000 population [2]. In Asia, it is more prevalent in a few regions of China, particularly Anhui province and Japan, due to genetic factors and consanguinity [3]. In recent studies from Korea and Morocco, prevalence is estimated to be 3.06/100000 and 3.88/ 100000 in the population respectively [4,5]. Often, diagnosing WD can be challenging due to its rarity, diverse clinical presentations, and lack of awareness of this treatable entity [6]. Recently, genetic tests have emerged as a valuable tool for diagnosing WD. While effective treatments are available for WD, each chelating agent needs careful monitoring. There is ongoing research on newer therapies and improved monitoring techniques for WD management [7]. In this narrative review, we will briefly discuss the clinical presentation of WD and its diagnosis and then elaborate on its management, focusing mainly on the recent changes or updates in the treatment with actual case scenarios.

A PubMed database search was done using the terms “Wilson Disease”, “Wilson’s Disease” and “Hepatolenticular degeneration”. Our search was restricted to Human subjects and English language articles, where the terms appeared in the title or the abstract. Articles published were searched, with special emphasis on those published in the last seven years, i.e., after 2017 till the date of search ((6th May 2024 and again on 5th July 2024), were considered for review.

The search identified 1057 articles, which included 1035 full-text articles. Again, 720 articles were excluded due to lack of relevance, as they were unrelated primarily to WD or WD treatment. The remaining 315 articles were selected for further review. There were 199 review articles, of which 138 were mainly case reports and brief literature reviews and were excluded. The remaining 61 were selected. There were eight randomized controlled trials and 11 systematic reviews comprising hepatic, neural, skeletal, and other forms of WD. We also reviewed cross-references of selected publications for added perspective.

## Clinical presentation

Wilson’s Disease (WD) was first described by S A K Wilson in 1912; its classical picture included hepatic and neurologic abnormalities [8]. However, with advances in diagnostic techniques such as next-generation genetic sequencing and the broader availability of magnetic resonance imaging, unusual presentations of WD with atypical hepatic and neurological abnormalities are being diagnosed more efficiently, and also asymptomatic carriers, who are detected while screening family members of WD [9]. Sometimes, WD patients can also present with hepatic, neurological, skeletal, and hematological abnormalities, which can be identified with advanced genetic testing [9]. Patients with WD can present at any age, ranging from 3 to 55 years. Commonly, pediatric (aged <18 years) patients present with a hepatic form of WD, and adults can present with features of liver dysfunction along with features of brain involvement. However, isolated neurological presentation of WD is also observed both in pediatric and adult populations [10,11,12].

Neurological manifestations of WD are highly variable. Common presentations include dysarthria, gait abnormality, and dystonia, with a dystonic smile reflecting extrapyramidal system involvement [13]. Tremors are also observed in WD, often postural, rubral, and rest tremors based on the site of copper deposition. Other neurological symptoms include seizures, ataxia, and stiffness of limbs due to the involvement of the cerebral cortex and pyramidal system [14]. Depression and anxiety are also noted in WD and sometimes can precede the development of neurological manifestation [8,15]. Sensory symptoms and features of peripheral neuropathy are uncommon in WD. There is no clear association between specific neurological manifestations and *ATP7B* mutation subtypes in WD [16]. However, tremor predominance can be observed with missense variants. Hepatosplenomegaly with dystonia and rigidity is predominant in truncating variants [17].

Due to varied neurological presentations of WD, a detailed neurological examination is necessary for patients to identify various neuraxis involvements and perform clinical monitoring. Overall, for assessment, there are specialized rating scales such as Unified Wilson’s Disease Rating Scale (UWDRS) [18] and Global Assessment Scale (GAS) [19] for understanding and assessing the severity of neurological manifestation and also as an objective measurement of clinical progression during follow up.

## Traditional diagnostic workup

**Figure 1:**
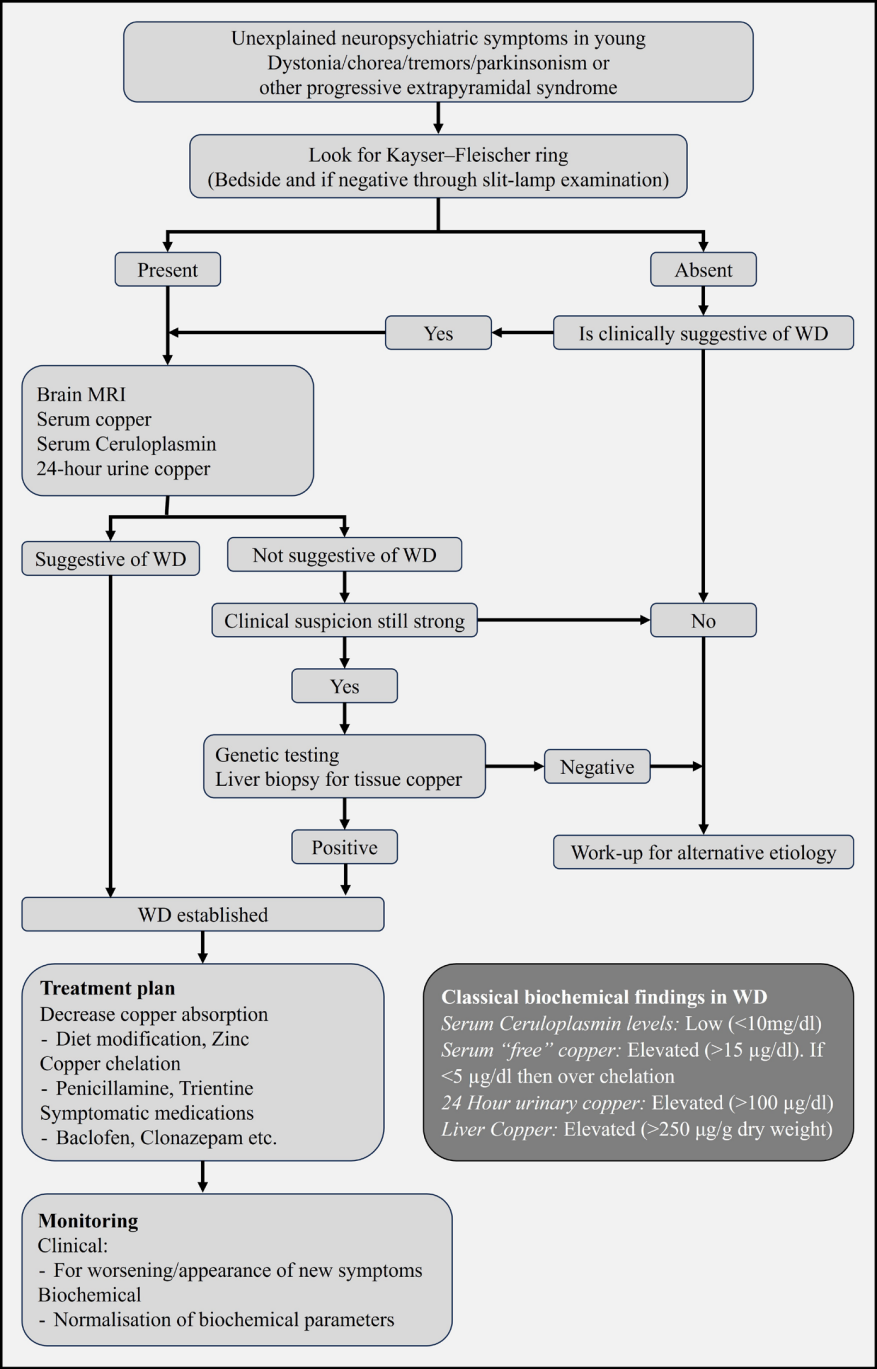
Algorithm showing the approach to a suspected Wilson’s Disease patient. MRI: Magnetic resonance imaging; WD: Wilson disease.

Diagnosing WD is often challenging due to complex clinical presentations and varying imaging findings (Figure 1). In addition to clinical features suggesting liver/ brain involvement, the Kayser–Fleischer ring (KF ring) in the cornea provides a clue to recognize patients with WD. Measurement of serum copper, ceruloplasmin levels, and urinary copper estimation helps diagnose WD. Overall, early diagnosis is essential to prevent complications.

### Serum Ceruloplasmin

Ceruloplasmin is an acute phase reactant and may be elevated in acute illnesses. Its level in serum is often low in patients with WD (Normal range of 30–50 mg/dL, which may vary between labs) [20]. It is determined using immunoassays, which measure holoceruloplasmin (copper-bound) and apoceruloplasmin (copper-free). Hence, the ceruloplasmin level may be expected in up to 1/3rd of patients with WD; mere normal ceruloplasmin levels do not exclude WD [6]. One of the recent studies has demonstrated that serum ceruloplasmin levels of less than 10mg/dL have a positive predictive value of 100% for WD [21]. Serum ceruloplasmin levels can be low in conditions other than WD. Common conditions of low serum ceruloplasmin include malabsorption syndromes, marked protein loss due to protein-losing enteropathy or non-selective renal loss, end-stage liver diseases, Menkes disease, and aceruloplasminemia [22]. In such cases, determination of the tissue copper levels and other clinical, biochemical, and radiological parameters can help establish a diagnosis of WD. As the ceruloplasmin gene has estrogen response elements, it can also be elevated during pregnancy, hormone replacement therapy, and while using a few oral contraceptive pills.

### Serum Copper

Due to the spillage of free copper from the damaged hepatocytes, serum-free copper is elevated in patients with WD. However, total serum copper in WD is usually below normal due to the decreased ceruloplasmin and, thereby, the reduced bound copper component of the total serum copper. (Normal: 63–140 μg/dl) Therefore, total serum copper levels alone may not be sufficient for diagnosing WD as they fluctuate. Even non-ceruloplasmin-bound free copper has been studied to diagnose WD. This non-ceruloplasmin-bound free copper can be estimated as the difference between the measured serum total copper levels in micrograms per deciliter and the calculated ceruloplasmin-bound serum copper by multiplying 3.15 by the serum ceruloplasmin concentration in milligrams per deciliter (Total serum copper (μg/dl) – (3.15 × ceruloplasmin) mg/dl). This is because the amount of copper associated with ceruloplasmin is approximately 3.15μg per milligram [23,24]. The normal range of this estimated free serum copper is 15–25 μg/dl. The major limitation of using the estimated non ceruloplasmin bound copper as a diagnostic test for WD depends on the adequacy of the methods for measuring serum ceruloplasmin and total copper. Its utility is more in treatment monitoring than in the diagnosis of WD. The ideal way is to measure the exact level of free serum copper, which is difficult and often unavailable in many regions [25].

### Urinary copper

24-hour urinary copper is often elevated (over 100 µg/day) in patients with WD, especially those with neuropsychiatric WD. It is one of the most sensitive screening tools for WD. The conventional cutoff taken as diagnostic of WD is >100 μg/24 h (>1.6 μmol/24 h) in symptomatic patients [26]. It usually does not exceed 40 µg/ day in asymptomatic heterozygous variant carriers. False positive results may be seen in severe liver failure due to other causes [27]. This test is used widely to diagnose WD but is cumbersome and challenging to collect. It is essential to ensure that the urine is collected in an appropriate copper-free container and that complete urine over 24 hours is collected rather than calculating the estimated 24-hour urine copper by measuring the concentration of a single-time urine sample.

In addition to its utility in diagnosing, urinary copper excretion is essential for monitoring and modifying treatment in patients with WD. However, during treatment monitoring, for a patient on penicillamine, the urinary copper excretion should be measured after withholding penicillamine for 48 hours to accurately measure natural copper excretion without the influence of medications [28]. Another utility of 24-hour urine copper is with the penicillamine challenge test. This test assesses the 24-hour urine copper after challenging the patient with 740–1000 mg of penicillamine. However, many of the reported results of this test utilized different dosages and timing for administration of the penicillamine and did not have high clinical utility. With the advent of better investigations, including genetic testing, this test is not currently advised for all patients.

### Brain imaging

Computed Tomography (CT) scan of the brain is the initial brain imaging done in most places due to wide availability. However, it is less sensitive to detect changes observed in WD. Typical findings include cortical and basal ganglia atrophy. Brain MRI is more practical and sensitive than a CT scan in diagnosing WD. Common changes include signal changes in the thalamus, basal ganglia, pons and atrophy of brain parenchyma [29,30]. T2 signal changes in the midbrain, “face of the giant panda,” and “bright claustrum” signs are pathognomonic of WD in an appropriate clinical setting. However, these changes are observed only in 10–15% of cases [31]. In some patients, MRI can show features of cerebral atrophy and non-specific white matter signal changes. Imaging is used mainly to aid in diagnosing WD. It is rare to have normal MRI imaging in a patient with neurological WD, so a normal MRI essentially rules out a possibility of neurological WD. The follow-up MRIs do not help monitor the response to the treatment as clinical and biochemical improvement may not always translate to improvement in the underlying structural/imaging changes [32]. In most cases, there is a correlation between clinical and radiological improvement. However, a few patients may have clinical improvement despite radiological worsening and vice versa. Recently, many new MRI techniques, such as quantitative susceptibility mapping and software like volumetric FreeSurfer, have been studied to look for treatment responses in WD [33].

## Advanced genetic Testing

Now, WD has been observed in almost all ethnic groups across the globe [34,35,36]. The first description of genetic evaluation was in an Arab family in 1987, which identified multiple family members to be suffering from WD. The cause was identified as a mutation in the red cell enzyme esterase D. Later; several groups independently identified the WD gene, which consisted of a transcript of 7.5kB and was primarily expressed in the liver, kidney, and placenta. Over the last two decades, significant developments have been made in understanding the genetic aspect of WD [37]. Though the classical description of WD is of autosomal recessive inheritance, there are reports of two or more generations being affected in the same family with “pseudo-dominant” inheritance, highlighting not to exclude clinical susception in suspected patients of WD with just the pattern of inheritance and family history [38]. At the cellular level, the WD gene codes for a trans-Golgi P-type ATP transport protein [39]. This protein has multiple metal binding domains, one ATP binding domain, one cation channel, a phosphorylation region, and a transduction domain [40]. The protein functions to channel copper to the canalicular membrane of the hepatocyte to facilitate the excretion of excess copper via bile. Dysfunction of this protein leads to WD. Currently, ATP7B is the only known gene responsible for WD, and disease-causing variants have been reported in almost all exons of this gene [41].

More than 1200 disease-causing variants of the WD gene (ATP7B) have been detected, of which the majority are missense variants [42,43,44]. The most common are p.H1069Q in Europe and the North American region and p.R778L in the East Asian population [41]. Most patients are in a compound heterozygous state, with different variants on each allele. Some studies suggest that some variants are associated with the early onset of the disease [45]. Missense variants may cause tremors with slow progressive hepatic failure and neurologic deterioration. Truncating variants that lead to a lack of protein production may result in early and fulminant hepatic presentations. Truncating variants are predominantly associated with hepatosplenomegaly and extrapyramidal features, such as rigidity and dystonia [46]. R778L mutation is associated with earlier presentation and lower serum ceruloplasmin levels [47]. Variability in the presentation of the disease among family members suggests that other factors may influence disease presentation. However, no clear evidence exists to categorize genetic subtypes with their corresponding clinical phenotype [48].

Owing to the numerous disease-causing variants, high-throughput Next Generation Sequencing (NGS) that can identify an abnormality in the whole gene is preferable to Sanger sequencing of a particular variant, exon, or part of the gene. Exome sequencing helps identify variants in all exons of ATP7B (coding regions of genes that directly translate to proteins)and adjacent introns (non-coding regions that are spliced out during RNA processing). For deep intronic variants and the promotor region variants (regulatory regions controlling gene transcription), target ATP7B sequencing can be done, spanning the whole gene [49]. Along with the index case, family members also may undergo genetic testing. The index case’s DNA is then used as a reference to compare and recognize the disease-carrying gene among the family members, which also helps in diagnosis. Once the disease-causing variant is identified in the patient, Sanger sequencing of these particular variants can be performed on the family members to determine the carriers and the affected or the unaffected family members.

Overall, the determination of serum ceruloplasmin levels and urine copper levels, supported with genetic testing, often aids in diagnosing WD. In addition, the Leipzig scoring system is used in diagnosing WD, which is based on a scoring system involving clinical, biochemical, and molecular testing variables. A score of ≥4 suggests WD, and ≤ 2 excludes WD. This score has been validated in children and adults [1,50]. In neonates, tandem mass spectrometry measuring ceruloplasmin levels in dried blood spots has been tried for WD screening [51,52].

### Other investigations

Liver function tests, Kidney function tests with urine analysis, a Hemogram with peripheral smear to look for features of anemia, X rays of limbs and joints to look for osteopenia are also used to look for other systems involvement, as young age and male status are associated with a higher risk of osteopenia [53]. In addition to these routinely used diagnostic tests, a few other tests are used to diagnose WD. Some of them include serum copper/ceruloplasmin ratio (µg dl^-1^) of more than 2 to distinguish a patient with WD from a healthy subject or a heterozygote (ratio <1) [54]. Another test is the relative exchangeable copper (ratio of serum exchangeable copper (CuEXC)/ total serum copper (CuT)), which, if more than 18.5%, suggests WD [54]. Radiocopper assay, based on the principle of decrease in radiocopper incorporation into ceruloplasmin in WD, is also used in WD diagnosis [55]. However, this test is not widely available. Measurement of copper concentration in liver biopsy specimens is also one of the methods of diagnosis and is still considered the gold standard. However, this is an invasive procedure. Its main advantage lies in measuring quantitative copper levels, as most WD patients have >250 µg/g (Normal: <50 µg/g) of dry weight and looking simultaneously at the stage and grade of liver injury. In selected cases, follow-up estimation of copper helps in treatment monitoring [56,57].

## Management

Treatment options in WD have changed rapidly, especially in the last few decades [56,58]. Often used treatments with well-established evidence in WD are copper chelators, medications for symptom relief, and definitive treatment, i.e., liver transplantation in patients with fulminant liver failure [59]. However, modern therapy often involves a combination of lifestyle modifications and genetic counseling, in addition to medications used in WD [60,61,62]. Recently, many studies have been conducted regarding the role of gene therapy and targeting *ATP7B* gene expression. Given the rarity of the disease, it is also difficult to conduct extensive, double-blind, placebo-controlled studies to compare treatment regimens in WD [56]. In general, monitoring and treatment optimization in WD also remains complex. The treatment protocol varies for hepatic and neurological disease, and this review mainly focuses on neurological WD management. Furthermore, measuring outcomes uniformly is also tricky owing to the varied presentations of WD [9,56]. The medications used in WD are described below (Tables 1 and 2).

**Table 1:** Dose, mechanism of action and important points regarding commonly used chelating agents in Wilson’s disease.


MEDICATION	DOSE	MECHANISM OF ACTION	SIDE EFFECTS AND INTERACTIONS	REMARKS

D-penicillamine	20 mg/kg/day (rounded to the nearest 250 mg). Slow escalation (preferably 250 mg alternate day in the beginning followed by increments of 250 mg every week to the target dose)	Copper chelator-enhances copper excretion through kidneys	Abdominal pain, Diarrhoea, Skin rashes Pyridoxine (25–50 mg/day) supplementation due to its anti-pyridoxine effect.	To be administered 1 hour before or 2 hours after meals Paradoxical worsening Has anti-pyridoxine effect

Trientine	20 mg/kg/day (rounded to the nearest 300 mg)	Copper chelator-enhances copper excretion through kidneys	Nausea, Stomach pain, Joint pains Iron deficiency due to chelation effect	To be administered 1 hour before or 2 hours after meals Paradoxical worsening Monitor for iron deficiency

Zinc salts	150 mg/day of elemental zinc in three divided doses	Inhibits the intestinal uptake of copper by inducing enterocyte metallothionein	Generally, it is well tolerated. Can have stomach pain, nausea	Not to take with food or with copper chelating agents. To be given with adequate spacing. Can have gastritis features Safe in pregnancy


**Table 2:** Dose, mechanism of action and important points regarding commonly used symptomatic medications in Wilson’s disease.


MEDICATION	DOSE	MECHANISM OF ACTION	REMARKS

*Tremor*			

Propranolol	40–240 mg/day in divided doses	Nonselective beta receptor antagonist	Monitor heart rate and blood pressure

Clonazepam	0.5–4 mg/day	Direct effect on benzodiazepine receptor and facilitates GABAergic transmission in the brain	Can have excessive sedation with higher doses

Gabapentin	Start with a dose of 100–300 mg/d and slowly increase up to 1200–2400 mg/d in three divided doses or till tolerated	Action of presynaptic voltage-gated calcium channels and prevents the release of excitatory neurotransmitters	It may cause vision blurring, unsteadiness, and dizziness.

Primidone	Start 100–150 mg/d in 2–3 divided doses and titrate slowly up to 750 mg/d or till tolerated	Interacts with voltage-gated sodium channels and inhibits repetitive firing of action potentials	The rapid increase in dose can cause ataxia and depression.

*Dystonia*			

THP	Start with 1 mg/day, slowly increase 1 mg every 3–5 days until optimal response is achieved/ side effects emerge	Muscarinic acetylcholine receptor antagonist	Few children may experience chorea Monitoring for dry mouth, constipation, and urinary retention. Contraindicated in narrow-angle glaucoma

Baclofen	Start with 5 mg/day, slowly increase 5 mg every 3–5 days to reach a maximum dose of 60 mg/day based on clinical response and side effects	Presynaptic GABA receptor agonist	Abrupt withdrawal can cause psychosis or seizures

Botulinum toxin	25 to 200 units based on the type of dystonia and muscles involved.	Blocks the release of acetylcholine into the neuromuscular junction	Highly effective in focal and segmental dystonia. Can have transient weakness of injected muscles.

*Parkinsonism*			

Levodopa	Start with 100 mg of levodopa (with carbidopa combination), and slowly increase one tablet every 3–5 days to reach optimal response or a maximum of 1200 mg/day	Converted to dopamine in presynaptic terminals of dopaminergic neurons, which further acts on postsynaptic dopamine receptors.	Administered with a combination of carboxylase inhibitor carbidopa. Monitor for nausea orthostasis with initial doses

Amantadine	300 mg/day divided into 2–3 daily doses	The weak antagonist of NMDA-type glutamate receptors increases dopamine release and blocks dopamine reuptake.	Beneficial in patients with dyskinesias

*Chorea*			

Tetrabenazine	Start with a 12.5 mg dose once daily, and slowly increase by 12.5 mg every 3–5 days to a target of 25–100 mg daily.	Depletes vesicular stores of dopamine by inhibiting the monoamine transporter-2	Rapid increase in dosage can cause drowsiness, parkinsonism, depression, anxiety, and akathisia.


GABA: Gamma amino butyric acid; NMDA: N-methyl D-aspartate; THP: Trihexyphenidyl

### Copper chelation therapy

#### D-Penicillamine

Historically, penicillamine’s copper-depleting effect was recognized and adopted as the first chelator of choice in treating excess copper in WD patients [63]. Studies have also shown that D-penicillamine can reverse the hepatic, psychiatric, and neurologic manifestations of WD [64]. Hence, it is considered the gold standard for treating WD and serves as the yardstick against which the efficacy of other treatment modalities is measured. Asymptomatic patients can be treated effectively and for prolonged periods without significant treatment-related complications [50]. It is also effective in treating patients with severe liver dysfunction without any features of encephalopathy [65].

D-penicillamine increases copper excretion through urine and induction of endogenous intracellular metallothionein, reducing absorption and promoting copper elimination in feces. The dosage is 1 to 2 g per day in 4 divided doses, 30 minutes to 1 hour before or 2 hours after meals, as food reduces D penicillamine absorption by about 50% [66]. The dose is calculated in children as 20 mg/kg/day, rounded to 250 mg [67]. Slow escalation of Penicillamine dose is recommended to avoid acute paradoxical neurological deterioration. The penicillamine dose needs to be reduced if the paradoxical neurological decline is observed in patients compliant to penicillamine. As the worsening stabilizes, penicillamine should preferably be substituted with an alternative available chelating agent such as trientine. In case an alternative option is not available or not accessible, penicillamine can be reintroduced at an even slower dose escalation rate and with strict clinical monitoring. Pyridoxine supplementation at 25 mg is used to counteract the weak anti-pyridoxine effect of D-Penicillamine, though studies have not shown a clear pyridoxine deficiency state [68]. Sometimes, it becomes difficult to differentiate between the natural time course of WD and treatment-related early deterioration [69].

While on D-Penicillamine, the patient is monitored using non-ceruloplasmin-bound plasma copper levels, which should be more than 25µg/dl at the start of treatment and reduce to 15–25 µg/dl during maintenance therapy. Also, the 24-hour urinary copper excretion should be 500–1000 µg per 24 hours at the beginning of the treatment and should reduce to 200–500 µg per 24 hours during maintenance therapy [54]. Maintenance therapy must be continued for a lifelong period to prevent the re-accumulation of dietary copper in the body [70]. During maintenance therapy, one should also monitor serum-free copper levels not to go below 15 µg/dl. Low 24-hour urinary copper suggests over-chelation, copper deficiency, and hypocupraemia-associated myelopathy in such cases. Also, if there are any new onset pyramidal signs during the maintenance phase, one should always consider the possibility of over-chelation and hypocupraemia-associated myelopathy [70].

#### Trientine

Triethylene tetramine dihydrochloride is an alternative to D-Penicillamine for intolerant patients due to penicillamine-associated arthritis and renal tubular necrosis [71,72]. It is also a preferred chelating agent in WD patients with associated thrombocytopenia and neutropenia secondary to splenomegaly. Owing to its fewer side effects, some physicians consider it a first-line drug. Like D Penicillamine, it should also be administered an hour before or 2 hours after meals. Its exact mechanism of action is not known. The recommended dose is 750–1500 mg daily in 3 doses [73]. In children, the recommended dose is 20 mg/kg/day (rounded to the nearest 300 mg) [67]. The major side effect is the development of anemia due to copper deficiency [74,75]. This drug must be stored at a temperature of 2 to 8°C and needs a cold chain while transporting the medicine. Similar to penicillamine, trientine can also lead to neurological deterioration after treatment initiation [76] and symptoms are often controlled with dose reduction [77]. Sometimes, these neurological deteriorations require the use of steroids and rarely with the replacement of trientine with other chelating agents for controlling the symptoms [56].

#### Thiomolybdates

Tetrahydromolybdate is an experimental drug yet to be approved by the FDA. Though veterinarians initially used it for the treatment of copper poisoning in animals, this drug is still under investigation for human use and is not commercially available. It has copper chelating and antiangiogenic properties. It also acts by induction of intestinal metalloproteins and copper chelation [9]. In contrast to the other chelating agents, Thiomolybdate is administered with meals. It can be safely used in patients with severe neurological manifestations as it does not cause neurological worsening on treatment initiation [78]. Elevation of transaminases can occur with bone marrow suppression as a side effect.

#### Zinc

Zinc is given orally as zinc salts and is absorbed by intestinal cells. It primarily induces metallothionein in the intestine and liver, sequestering copper. However, zinc monotherapy has yet to be established as its de-coppering effect has not been studied since most patients have already received other chelating agents. Guidelines also have recommended starting a chelating agent such as D-penicillamine or Trientine early on in symptomatic patients [50]. Maintenance therapy can be done with reduced doses of zinc or other chelating agents. If a patient has an intolerance to both D-penicillamine and trientine, zinc monotherapy can be used [79]. Zinc can also be given as first-line therapy in asymptomatic individuals of WD [79]

When a patient is on zinc therapy, the aim is to reduce the urinary copper excretion to less than 100 µg per 24 hours at the beginning of treatment and to 30–80 µg per 24 hours during maintenance therapy, with the non-ceruloplasmin bound copper levels being normal in the plasma. Urinary zinc excretion levels above 1.5–2 g per day indicate compliance with treatment [50]. These Zinc salts should not be combined with food or chelating agents; each needs to be spaced out for better effect [80]. Zinc salts are available in different formulations, such as zinc sulfate, zinc acetate, zinc citrate, etc. Zinc sulfate is a less expensive form but is least absorbed from the stomach and has the propensity to cause gastritis, which in turn can lead to poor compliance with medications by patients. Zinc acetate is less likely to cause gastritis and is better tolerated than zinc sulfate.

Dimercaprol (2,3-dimercapto-1-propanol), which was used in intramuscular injectable forms for WD before, has been replaced by oral chelators in recent times, and Dimercaptol is rarely used in recent days for WD [56].

### Symptomatic therapy

#### Medical therapy

There are various medications which are used for are used for symptomatic relief (Table 2). For tremors, beta-blockers like propranolol and benzodiazepines are tried in refractory cases with disabling tremors; neurosurgical treatment with thalamotomy has also shown promising results [81]. For dystonia, anticholinergics and antispastic medicines like baclofen, benzodiazepines/ clonazepam are often tried [82]. Botulinum toxin can also be effectively used for focal dystonia; however, it may require repeated injections [83]. Dopaminergic medications and amantadine are tried for parkinsonism in WD with promising results [82,84]. Antiseizure drugs and psychiatric drugs may also be needed in patients with seizures based on semiology and type of behavioral symptoms, respectively. Tetrabenzine has been tried in chorea with mixed results [84].

#### Surgical therapy

Deep Brain Stimulation is tried in patients with selected cases of refractory tremors and dystonia, targeting the ventral intermediate nucleus of the thalamus and globus pallidus interna, respectively, with promising results [85,86]. In patients with disabling dysphagia, neuromuscular electric stimulation has shown beneficial results [87]. Along with copper chelators and symptomatic medications, a comprehensive approach with multidisciplinary care with speech therapy, physiotherapy, and occupational therapy often leads to good clinical outcomes.

### Management of asymptomatic individuals

Treatment is essential even for asymptomatic individuals, detected during WD screening, to prevent disease progression. The mainstay of treatment for asymptomatic WD includes Zinc salts with chelators at lower doses than those used in symptomatic WD individuals. These asymptomatic patients are also advised for regular follow-ups to monitor copper levels and liver function. Overall, early diagnosis and treatment are key for preventing disease progression of WD [88].

### Case vignettes

**Figure 2:**
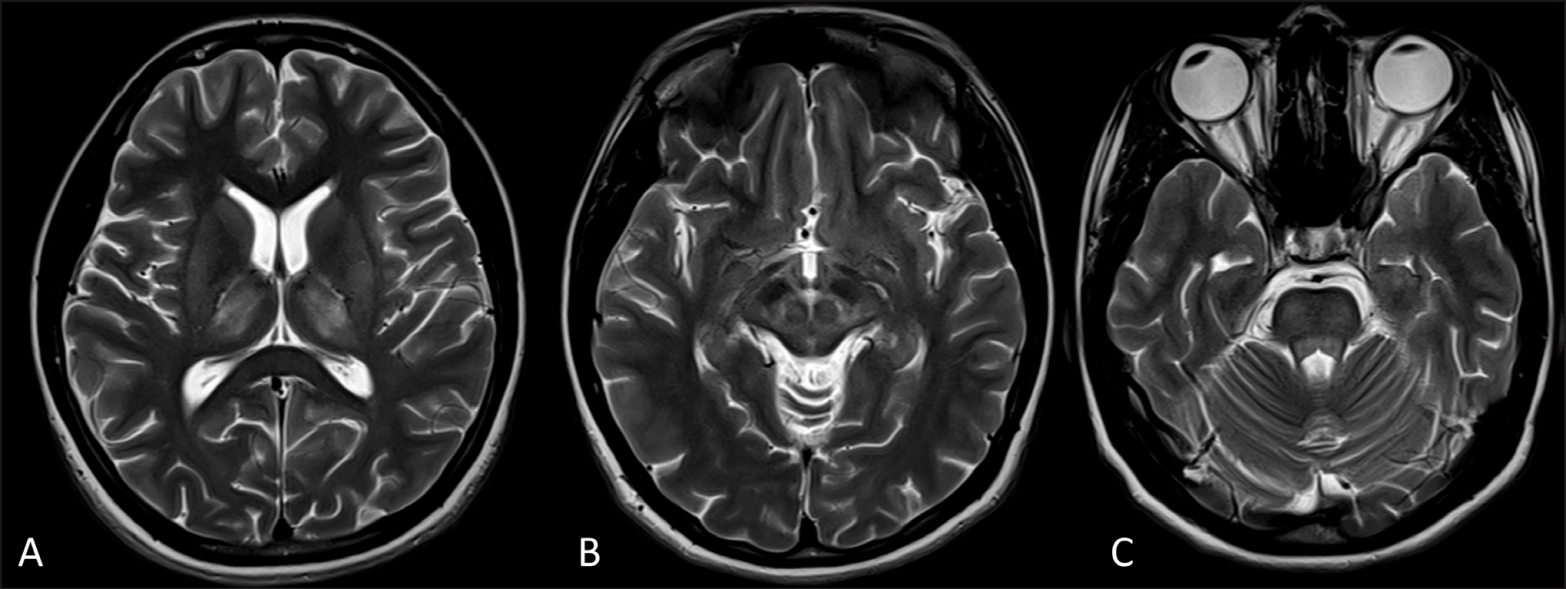
A to C- MRI axial sections of Case-1 depicting T2 hyperintensities in bilateral thalamus **(A)**, face of the giant panda sign in the midbrain **(B)**, and face of the miniature panda sign in the pons **(C)**. MRI: Magnetic resonance imaging.

**Case-1:** A 10-year-old boy, born to non-consanguineous parentage, with a history of recurrent jaundice since the age of 8 years, presented with abnormal posturing of the left upper and lower limb for the last 4 months, which progressed over two months to involve the right upper and lower limb associated with speaking and swallowing difficulty. On examination, he had a K-F ring in both eyes, with generalized dystonia, dysarthria, and dystonic smile (Video–1). His serum total copper (30.8 mg/dL; lab normal range: 70–150) and ceruloplasmin (<3.0 mg/dL; lab normal range: 15–30) levels were low, and 24-hour urinary copper was high (352 μg/24 hours; normal range: <70 μg/24 hours). His MRI showed T2 hyperintensities in the bilateral thalamus (Figure 2A), midbrain (Face of giant panda sign, Figure 2B), and pons (face of miniature panda sign, Figure 2C) consistent with WD diagnosis. Ultrasound of the abdomen showed features of chronic liver disease with no splenomegaly or evidence of portal hypertension. He was diagnosed to be having WD and was initiated on treatment. D-penicillamine was initiated along with a zinc acetate tablet, along with symptomatic therapy consisting of levodopa/carbidopa, trihexyphenidyl, and clonazepam.

**Video 1:** Video of Case-1 showing oro-mandibular dystonia, sialorrhea, distal predominant dystonia in all four limbs, lingual chorea, dysarthria, and worsening of dystonia on walking. The video was taken after written informed consent was obtained for video recording and publication in print and online.

His elder sister was clinically asymptomatic. Ophthalmological examination under slit limp was negative for the K-F ring. Her serum copper (114.6 mg/dL; lab normal range: 70–150), serum ceruloplasmin (23.1 mg/dL; lab normal range: 15–30), and 24-hours urinary copper was high (17 μg/24 hours; normal range: <70 μg/24 hours) were within normal limit.

The parents wanted to know the children’s underlying genetic carrier status, so exome sequencing was performed. The patient carried two pathogenic variants (NM_000053:c.174dup; p.Thr59HisfsTer19 and NM_000053:c.3445G>A;p.Gly1149Arg) in the *ATP7B* gene in compound heterozygous trans state confirming the diagnosis of WD genetically as well. The asymptomatic elder sister with no clinical and biochemical evidence of WD had only the NM_000053:c.3445G>A;p.Gly1149Arg variant in heterozygous state confirming the unaffected carrier state in her. The family was counseled regarding the same.

**Case-2:** A 33-year-old male, born to consanguineous parentage, with normal perinatal and developmental history, presented with bilateral upper limb tremors for 7 years. It was present during rest and posture, hampering his daily activities. There were other significant neurological or systemic symptoms, including recurrent jaundice. On examination (Video–2), a KF ring was present in both eyes, with bilateral upper and lower limb tremors (rest, posture with wing-beating quality) and dystonic posturing of both upper limb fingers. Serum ceruloplasmin (8.9 mg/dL; normal: 15–25 mg/dL) and total serum-copper was low (35.4 μg/dL; normal: 70–150 μg/dL), and 24-hour urinary copper was elevated (135 μg/24 hours; normal: <70 μg/24 hours). Brain MRI showed signal changes in bilateral caudate, putamen, midbrain, and dorsal pons. Diagnosis of WD was made, and the patient was initiated on symptomatic treatment for tremors along with slow escalation of zinc acetate (final dose: 150 mg/day) and D-penicillamine (final dose: 500 mg/day). He showed a 40–50% improvement at the 6-month follow-up. This case was previously reported [89].

**Video 2:** Video of Case-2 showing both upper and lower limb tremors (rest, posture with wing-beating quality) with dystonic posturing of both upper limb fingers. The video was taken after written informed consent was obtained for video recording and publication in print and online.

**Case-3:** A 20-year-old lady presented with severely disabling and medically refractory tremors of 4 years duration (Video–3). There were no other significant neurological or systemic symptoms. On examination, a KF ring was present in both eyes along with the upper limb’s predominant bilateral wing-beating rubral tremor. On evaluation, serum ceruloplasmin (4.7 mg/dL; normal: 15–25 mg/dL) and total serum-copper was low (16.7 μg/dL; normal: 70–150 μg/dL), and 24-hour urinary copper was elevated (838 μg/24 hours; normal: <70 μg/24 hours). Diagnosis of WD was made, and there was not much improvement over the next two years despite being on the optimal doses of penicillamine, zinc, clonazepam, levodopa-carbidopa, and propranolol. Subsequently, she underwent a left ViM thalamotomy and had a remarkable improvement in tremors. Later, she was continued only on decoppering agents and the symptomatic treatment was discontinued slowly. This case was previously reported [74].

**Video 3:** Video of Case-3. *Segment-1*: Pre-operative video showing disabling rubral tremor in right upper limb with conducted tremor in left upper limb. *Segment-2*: 6-month follow-up video showing significant improvement of the tremor and functional ability after left ventral intermediate nucleus thalamotomy. The video was taken after written informed consent was obtained for video recording and publication in print and online.

**Case-4:** A 16-year-old girl, born of a non-consanguineous parentage, presented with complaints of speech disturbances for 8 months, tremulousness of both upper limbs for 7 months, abnormal posturing of all four limbs with gait difficulty for 5 months. For these above-mentioned complaints, she was evaluated at a nearby place and was diagnosed with Wilson disease. She was started on Penicillamine 250 mg once daily for 2 weeks, followed by 250 mg twice daily for 2 weeks. After one month of treatment, she developed sustained posturing of both upper limbs with an extension of both hands backward. She also developed flexion of the elbow and flexion of her hands, striatal toe, slowness of gait, and started having falls. On examination, she had a dense KF ring and mask-like facies, with a vacuous smile and drooling saliva. There was severe rigidity in the neck, with contractures in the left shoulder, elbow, wrist, fingers, and knee. She also had lower facial tremors and dystonia. Action dystonia of the right hand, bilateral feet, and toes was present. Investigations showed low serum ceruloplasmin and copper levels. Given the worsening of her neurological symptoms with the initiation of penicillamine therapy, the possibility of penicillamine-induced neurological worsening was considered, and her symptoms stabilized after reducing the dose (Video 4).

**Video 4:** Video of Case-4 showing the presence of rest and postural tremors in a right upper limb with generalized dystonia with a dystonic smile and striatal toe in the left leg. The patient had presented to us with a worsening of her symptoms after initiating D-penicillamine. At the 3-month follow-up, there is a reduction of tremors after reducing the D-penicillamine dose with the persistence of dystonia and dystonic smile. The video was taken after written informed consent was obtained for video recording and publication in print and online.

### Other therapeutic modalities

In addition to the above treatment, novel chelating drugs, targeted molecular therapies, and gene therapies are being studied or utilized.

Various newer medications like ammonium tetra molybdate, Methanobactin, Compound DPM-1001, and Chel2 have been tried in animal models and small clinical trials for assessing their efficacy in WD [90,91]. However, clinical utility is limited due to the lack of robust evidence.

In WD, stem cell therapy has also been tried to correct copper metabolism with mesenchymal/ embryonic stem cells [92]. Recently, Atp7b mRNA-expressing transplanted cells infused in the liver of WD patients [92].

### Liver transplantation

Liver transplantation is a definitive treatment to correct the hepatic metabolic defect in WD. It is a life-saving measure reserved for patients with fulminant hepatic failure. It is also recommended in patients with continuing worsening of hepatic dysfunction despite best medical treatment. Hepatic manifestations get reversed after liver transplantation, but the neurologic and psychiatric manifestations may not improve [9]. In a minority of cases, neurological worsening can occur post-liver transplantation [93,94,95]. Hence, in patients with severe neurological symptoms without significant liver dysfunction, liver transplantation is not recommended unless pharmacological de-coppering measures are tried [96].

Plasmapheresis is another treatment option considered in WD for reducing serum copper levels. It is also used as bridge therapy in patients who are awaiting liver transplantation and as rescue therapy in patients with fulminant disease [97].

Another treatment that has been considered in recent times is hepatocyte transplantation. It was performed on Long-Evans Cinnamon rats, which are good models for WD. The results show normalization of histological changes, indicating the potential of transplanted cells to repopulate and regenerate in the liver and normalize biliary copper excretion [98].

### Molecular and gene therapy

Molecular therapy targeting *ATP7B* and its function has also been tried in WD [99]. *However*, its clinical utility is still being researched. Gene therapy in WD is being attempted with the hypothesis of targeting monogenic disease with impaired biallelic dysfunction and aims to maintain *ATP7B* expression. It has been studied by delivering truncated *ATP7B* into rat models through adeno-associated vectors [100]. One of the significant challenges is the limited integration of ample ATP7B genetic information into viral vectors, and no promising clinical studies have been seen yet [101]. Clustered Regularly Interspaced Short Palindromic Repeats (CRISPR) targeted genomic editing for WD is also under research in animal models [102].

Transplantation of *ATP7B* mRNA-expressing cells into the liver is also tried to restore the standard functional capacity of the liver [92]. However, a significant limitation is the requirement of at least 40% of normal functioning cells in the liver to normalize copper metabolism, which limits the utility of this technique in WD patients [103].

## Treatment Monitoring and other management issues

The main goal of treatment monitoring is to stabilize clinical progression and reversal of biochemical abnormalities with adequate pharmacotherapy and to look for drug-induced adverse effects. Regular tracking of copper levels in blood and urine, along with monitoring of liver functions, is essential to assess the effectiveness of treatment and educate the family regarding treatment adherence [104]. It is also more critical to monitor patients who are on D penicillamine and trientine, which can cause paradoxical neurological worsening [76]. At each follow-up, clinical evaluation should be done to assess the worsening of any pre-existing neurological symptoms or the appearance of any new symptoms. General examination at follow-up should also focus on evaluating any drug-induced adverse effects—laboratory testing to look for liver dysfunction and monitor serum copper and ceruloplasmin levels. Guidelines suggest monitoring at least twice a year and more frequently in the initial period of diagnosis and treatment. In most cases, treatment is lifelong, and with very few exceptions, in patients following liver transplantation, it can be stopped early [105].

### Dietary modifications

Patients with WD are advised to follow a low-copper diet. It involves avoiding copper-rich foods like shellfish, liver, nuts, chocolates, vegetables like asparagus, potato with skin, and soybean sprouts. Nutritional supplements and healthy foods should be screened for copper contents [106]. In addition, zinc supplements are recommended, which inhibit copper absorption from the intestine. Overall, copper intake should be restricted to <0.9 mg/day. Even unlined copper mixing bowls and water jugs should be avoided by patients with WD.

### Screening of family members

**Figure 3:**
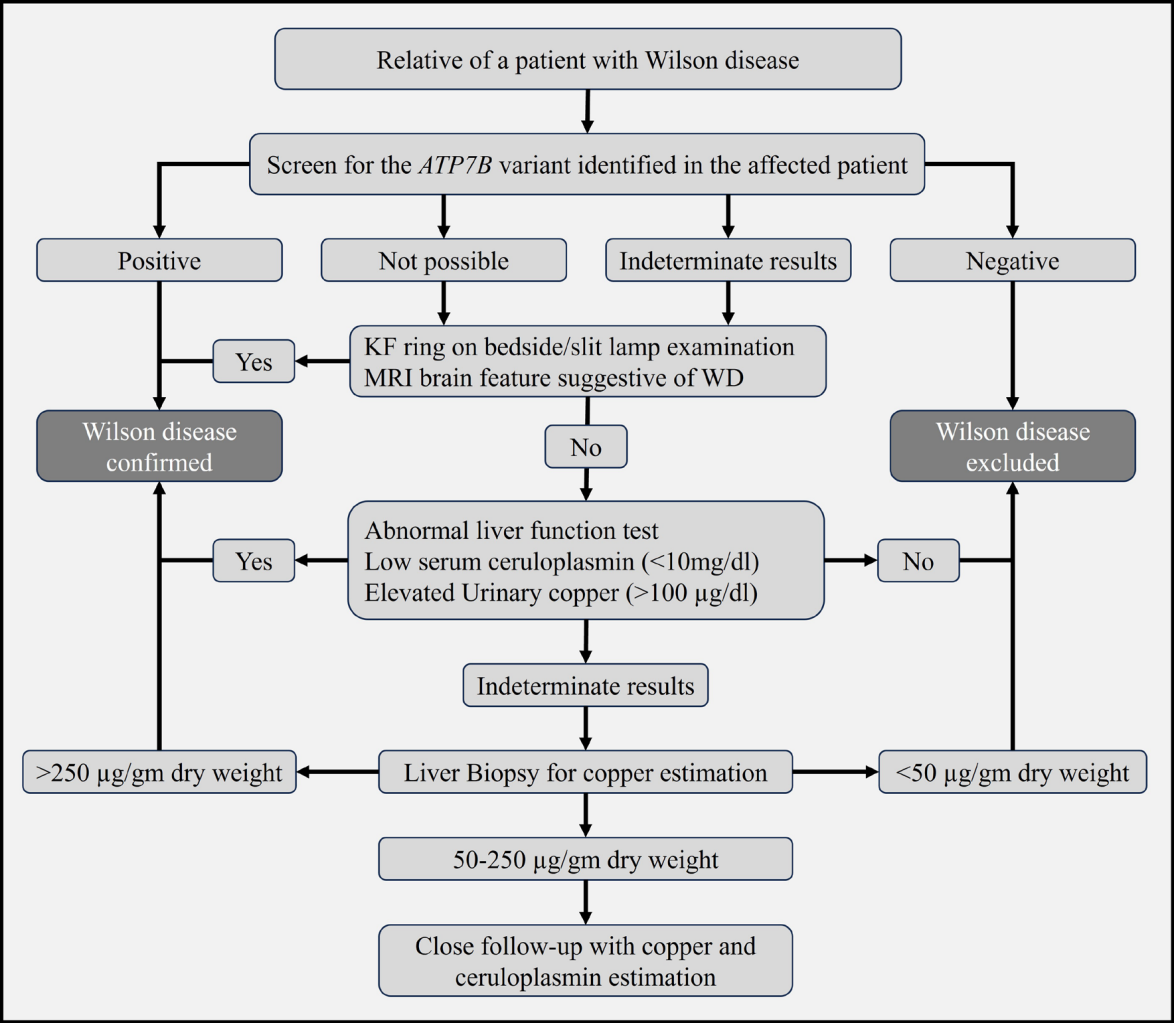
Algorithm for screening of asymptomatic siblings of Wilson’s Disease patient. KF ring: Kayser–Fleischer ring; MRI: Magnetic resonance imaging; WD: Wilson disease.

Screening of first-degree relatives of patients with WD is crucial for identifying asymptomatic individuals. Screening mainly includes history regarding prior jaundice, liver dysfunction, and neurological and psychiatric presentations (Figure 3). In addition, a complete neurological examination with an ophthalmological examination for KF ring, biochemical testing with serum copper, and ceruloplasmin estimation must be done on all these individuals. Suppose any of these observations are suggestive of WD. In that case, treatment must be initiated and closely monitored for any evidence of WD, even among presymptomatic individuals at the time of screening [107].

### Genetic counseling

Genetic counseling is recommended for WD patients and their family members to understand the genetic basis of the illness, risk stratification to other family members, and planning pregnancy. It is best coordinated through a genetic counselor. For pregnant women whose children are at risk of WD, prenatal testing is advised, and it is also vital to discuss preimplantation genetic testing measures to confirm if the fetus has the disease. Genetic counseling should also be offered to couples planning for pregnancy if there is a history of Wilson disease in their family to educate them regarding heterozygous carrier state and options of prenatal genetic diagnosis.

## Conclusion

Overall, WD is one of the treatable genetic disorders of copper metabolism. Recently, various atypical presentations have been observed in WD. Various phenotypes and genotypic associations have come to light with advances in genetic testing and a better understanding of WD. Many therapeutic agents have been tried to improve the management of WD; however, many newer agents are still being studied.
